# Supplementary material for: Mitochondrial Ca2+ uptake by the MCU facilitates pyramidal neuron excitability and metabolism during action potential firing
Source: Commun Biol. 2022 Sep 2;5:900. doi: 10.1038/s42003-022-03848-1 (PMC9440007; doi:10.1038/s42003-022-03848-1)
Supplement: Supplementary file 2 — Supplementary Information [file 42003_2022_3848_MOESM2_ESM.pdf]

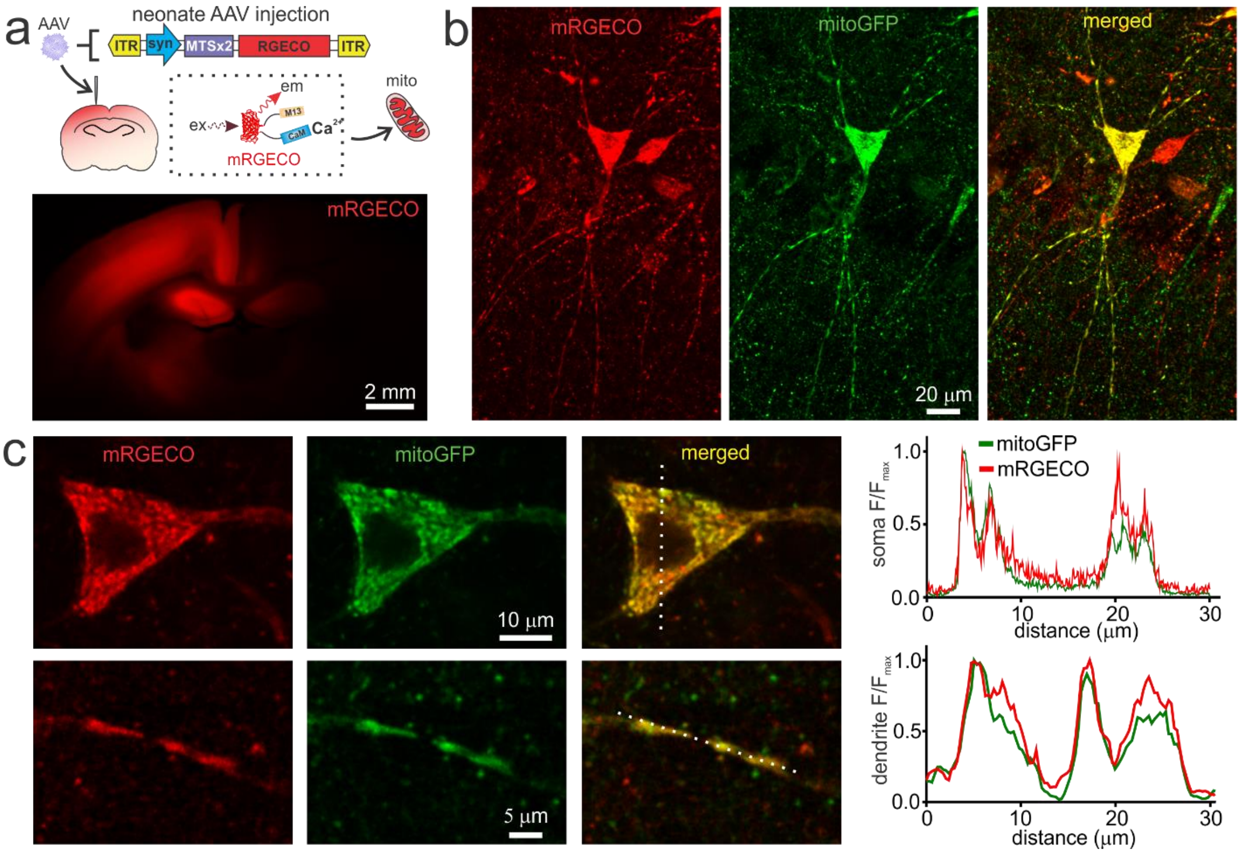

Supplementary Figure 1: Expression of mRGECO, a red fluorescent mitochondrial Ca^{2+} sensor, in rat pyramidal neurons *in situ*.

a) *Upper*, Neuronal expression of mRGECO, a mitochondrially-targeted Ca^{2+}-sensitive red fluorescent protein, was mediated by intracranial AAV delivery into neonatal rats. mRGECO was expressed under the control of the synapsin promoter (syn) and targeted to the mitochondria by two copies of a mitochondrial targeting sequence (MTS). *Lower*, Brain slice showing mRGECO fluorescence in the cortex and hippocampus after unilateral AAV injection. **b)** Maximum intensity projection of a hippocampal pyramidal neuron expressing mRGECO and mitoGFP in an acute brain slice. **c)** *Left*, mRGECO shows a punctate and tubular fluorescence pattern in the soma and dendrites which colocalizes with the mitochondrial morphology marker, mitoGFP. *Right*, Normalized fluorescence of mitoGFP and mRGECO in the soma and dendrite measured from the line plots depicted in the left panel.

Description of Additional Supplementary Information

File Name: Supplementary Video 1.

Description: *In situ* measurement of cytosolic and mitochondrial Ca^{2+} in a pyramidal neuron during action potential firing. *This video is related to experiments in Figure 2.* Time lapse video of a patch-clamped cortical pyramidal neuron expressing GCAMP6f and mRGECO to measure cytosolic and mitochondrial Ca^{2+} simultaneously. Neurons delivered a 50-Hz, 4 sec train of action potentials (stim) show rapid cytosolic Ca^{2+} transients while mitochondrial Ca^{2+} uptake occurred with a delay and slowly recovered following stimulation. The elapsed time (sec) relative to the stimulus onset is depicted in the upper left portion of the video.

File Name: Supplementary Video 2.

Description: Blocking the MCU with Ru360 causes a substantial enhancement in the magnitude and duration of the evoked cytosolic Ca^{2+} signal in pyramidal neurons. *This video is related to experiments in Figure 6.* Time lapse video of cytosolic Ca^{2+} measured from pyramidal neurons *in situ* with and without Ru360. Intracellular Ca^{2+} was measured with Fluo5N. Rainbow pseudocolouring depicts Fluo5N $\Delta F/F_o$ evoked by a 50 Hz, 4 sec action potential train (stim). Neuronal outline is shown in white and was determined using intracellular Alexa594 fluorescence. The elapsed time (sec) relative to the stimulus onset is depicted in the right portion of the video.
