# Supplementary material for: Mitochondrial Ca2+ uptake by the MCU facilitates pyramidal neuron excitability and metabolism during action potential firing
Source: Commun Biol. 2022 Sep 2;5:900. doi: 10.1038/s42003-022-03848-1 (PMC9440007; doi:10.1038/s42003-022-03848-1)
Supplement: Supplementary file 3 — Description of Additional Supplementary Data [file 42003_2022_3848_MOESM3_ESM.pdf]

Description of Additional Supplementary Files

File Name: Supplementary Video 1.

Description: In situ measurement of cytosolic and mitochondrial Ca2+ in a pyramidal neuron during action potential firing. This video is related to experiments in Figure 2. Time lapse video of a patch-clamped cortical pyramidal neuron expressing GCAMP6f and mRGECO to measure cytosolic and mitochondrial Ca2+ simultaneously. Neurons delivered a 50-Hz, 4 sec train of action potentials (stim) show rapid cytosolic Ca2+ transients while mitochondrial Ca2+ uptake occurred with a delay and slowly recovered following stimulation. The elapsed time (sec) relative to the stimulus onset is depicted in the upper left portion of the video.

File Name: Supplementary Video 2.

Description: Blocking the MCU with Ru360 causes a substantial enhancement in the magnitude and duration of the evoked cytosolic Ca2+ signal in pyramidal neurons. This video is related to experiments in Figure 6. Time lapse video of cytosolic Ca2+ measured from pyramidal neurons in situ with and without Ru360. Intracellular Ca2+ was measured with Fluo5N. Rainbow pseudocolouring depicts Fluo5N $\Delta F/Fo$ evoked by a 50 Hz, 4 sec action potential train (stim). Neuronal outline is shown in white and was determined using intracellular Alexa594 fluorescence. The elapsed time (sec) relative to the stimulus onset is depicted in the right portion of the video.

File Name: Supplementary Data 1

Description: The source data behind the graphs in the paper.
