# Supplementary material for: Mitochondrial Ca2+ uptake by the MCU facilitates pyramidal neuron excitability and metabolism during action potential firing
Source: Commun Biol. 2022 Sep 2;5:900. doi: 10.1038/s42003-022-03848-1 (PMC9440007; doi:10.1038/s42003-022-03848-1)
Supplement: Supplementary file 7 — Reporting Summary [file 42003_2022_3848_MOESM7_ESM.pdf]

nature portfolio

Corresponding author(s): Brian A. MacVicar

Last updated by author(s): Aug 12, 2022

Reporting Summary

Nature Portfolio wishes to improve the reproducibility of the work that we publish. This form provides structure for consistency and transparency in reporting. For further information on Nature Portfolio policies, see our Editorial Policies and the Editorial Policy Checklist.

Statistics

For all statistical analyses, confirm that the following items are present in the figure legend, table legend, main text, or Methods section.

n/a	Confirmed	
☐	☒	The exact sample size (*n*) for each experimental group/condition, given as a discrete number and unit of measurement
☐	☒	A statement on whether measurements were taken from distinct samples or whether the same sample was measured repeatedly
☐	☒	The statistical test(s) used AND whether they are one- or two-sided<br>*Only common tests should be described solely by name; describe more complex techniques in the Methods section.*
☒	☐	A description of all covariates tested
☐	☒	A description of any assumptions or corrections, such as tests of normality and adjustment for multiple comparisons
☐	☒	A full description of the statistical parameters including central tendency (e.g. means) or other basic estimates (e.g. regression coefficient) AND variation (e.g. standard deviation) or associated estimates of uncertainty (e.g. confidence intervals)
☐	☒	For null hypothesis testing, the test statistic (e.g. *F*, *t*, *r*) with confidence intervals, effect sizes, degrees of freedom and *P* value noted<br>*Give P values as exact values whenever suitable.*
☒	☐	For Bayesian analysis, information on the choice of priors and Markov chain Monte Carlo settings
☒	☐	For hierarchical and complex designs, identification of the appropriate level for tests and full reporting of outcomes
☒	☐	Estimates of effect sizes (e.g. Cohen's *d*, Pearson's *r*), indicating how they were calculated

Our web collection on statistics for biologists contains articles on many of the points above.

Software and code

Policy information about availability of computer code

Data collection	Images were acquired using Zen software (Zeiss). Neuronal membrane voltage and current were acquired using Clampex 10.2 software (Axon Instruments, Molecular Devices).
Data analysis	Electrophysiology data was analyzed using Clampfit 10.2 software (Axon Instruments, Molecular Devices). Image analysis was performed using ImageJ (v 1.53).

For manuscripts utilizing custom algorithms or software that are central to the research but not yet described in published literature, software must be made available to editors and reviewers. We strongly encourage code deposition in a community repository (e.g. GitHub). See the Nature Portfolio guidelines for submitting code & software for further information.

Data

Policy information about availability of data

All manuscripts must include a data availability statement. This statement should provide the following information, where applicable:

- Accession codes, unique identifiers, or web links for publicly available datasets
- A description of any restrictions on data availability
- For clinical datasets or third party data, please ensure that the statement adheres to our policy

The datasets generated during the current study are available from the corresponding author on reasonable request.

Field-specific reporting

Please select the one below that is the best fit for your research. If you are not sure, read the appropriate sections before making your selection.

☒ Life sciences ☐ Behavioural & social sciences ☐ Ecological, evolutionary & environmental sciences

For a reference copy of the document with all sections, see nature.com/documents/nr-reporting-summary-flat.pdf

Life sciences study design

All studies must disclose on these points even when the disclosure is negative.

Sample size	The number of replicates was not determined with power analysis prior to the start of the study. Sample sizes used are within the typical range observed in the literature for single neuron patch clamp experiments. These numbers allowed us to achieve reasonable power while also maintaining practical use of animal numbers and experimental resources.
Data exclusions	Data was rarely excluded from our study. When this was done, outliers were identified and removed based on analysis with the ROUT test in Prism or were clearly appropriate for exclusion as a result of noted experimental issues (e.g. loss of the integrity of the recording or cell in the brain slice preparation).
Replication	Data points for a given data set were collected from multiple animals over several weeks or months. We saw no apparent variation in the tested effects, indicating good replication.
Randomization	Experiments comparing control and test conditions were performed on discrete brain slices or neurons. The tissue sample or neuron used for each experimental condition was randomly selected.
Blinding	Blinding was difficult to perform during our experiments for practical reasons. Drugs had to be quickly made and administered through patch-clamp techniques that are technically challenging. Analysis was performed using reasonable practices to avoid influence by prior knowledge of the experiments.

Reporting for specific materials, systems and methods

We require information from authors about some types of materials, experimental systems and methods used in many studies. Here, indicate whether each material, system or method listed is relevant to your study. If you are not sure if a list item applies to your research, read the appropriate section before selecting a response.

Materials & experimental systems

n/a	Involved in the study	
☒	☐	Antibodies
☒	☐	Eukaryotic cell lines
☒	☐	Palaeontology and archaeology
☐	☒	Animals and other organisms
☒	☐	Human research participants
☒	☐	Clinical data
☒	☐	Dual use research of concern

Methods

n/a	Involved in the study	
☒	☐	ChIP-seq
☒	☐	Flow cytometry
☒	☐	MRI-based neuroimaging

Animals and other organisms

Policy information about studies involving animals; ARRIVE guidelines recommended for reporting animal research

Laboratory animals	Sprague Dawley rats were used for this study. Animals were 17-28 days old when experiments were conducted.
Wild animals	This study did not use wild animals.
Field-collected samples	This study did not use field-collected samples.
Ethics oversight	Animal experiments were performed in accordance with Canadian Council on Animal Care (CCAC) regulations and protocols approved by the University of British Columbia Animal Care Committee.

Note that full information on the approval of the study protocol must also be provided in the manuscript.
